# Author Correction: Functional characterization of a bioengineered liver after heterotopic implantation in pigs

**DOI:** 10.1038/s42003-021-02929-x

**Published:** 2021-12-08

**Authors:** Brett D. Anderson, Erek D. Nelson, DongJin Joo, Bruce P. Amiot, Aleksandr A. Katane, Alyssa Mendenhall, Benjamin G. Steiner, Aron R. Stumbras, Victoria L. Nelson, R. Noelle Palumbo, Thomas W. Gilbert, Dominique S. Davidow, Jeffrey J. Ross, Scott L. Nyberg

**Affiliations:** 1 Miromatrix Medical Inc, Eden Prairie, MN USA; 2 Department of Surgery, Mayo Clinic, Rochester, MN USA; 3 Department of Surgery, Yonsei University College of Medicine, Seoul, South Korea; 4 William J. von Liebig Center for Transplantation and Clinical Regeneration, Mayo Clinic, Rochester, MN USA

**Keywords:** Tissue engineering, Regenerative medicine

Correction to: *Communications Biology* 10.1038/s42003-021-02665-2, published online 7 October 2021.

In the original published version of the Article, the *y*-axis titles for Fig. 4d and Fig. 4e were incorrectly interchanged. The correct *y*-axis title for Fig. 4d is “Percent Survival” and the correct *y*-axis title for Fig. 4e is “Ammonia (mM)”. The error has been corrected in the PDF and HTML versions of the Article.

