# Supplementary material for: Functional characterization of a bioengineered liver after heterotopic implantation in pigs
Source: Commun Biol. 2021 Oct 7;4:1157. doi: 10.1038/s42003-021-02665-2 (PMC8497596; doi:10.1038/s42003-021-02665-2)
Supplement: Supplementary file 2 — Supplementary Material [file 42003_2021_2665_MOESM2_ESM.pdf]

Supplementary materials for

Functional Characterization of a Bioengineered Liver after Heterotopic Implantation in Pigs

Brett D. Anderson[1], Erek D. Nelson[2], DongJin Joo[2,3], Bruce P. Amiot[2], Aleksandr A. Katane[1], Alyssa Mendenhall[1], Benjamin G. Steiner[1], Aron R. Stumbras[1], Victoria L. Nelson[1], R. Noelle Polumbo[1], Thomas W. Gilbert[1], Dominique S. Davidow[1], Jeffrey J. Ross[1], Scott L. Nyberg[2,4,#]

[1]Miromatrix Medical Inc, Eden Prairie, MN, USA

[2]Department of Surgery, Mayo Clinic, Rochester, MN, USA

[3]Department of Surgery, Yonsei University College of Medicine, Seoul, South Korea

[4]William J. von Liebig Center for Transplantation and Clinical Regeneration, Mayo Clinic, Rochester, MN, USA

[#]Corresponding author:

Scott L. Nyberg, MD, PhD
William J. von Liebig Center for Transplantation and Clinical Regeneration - Mayo Clinic
200 First Street Rochester, MN 55905

Nyberg.Scott@mayo.edu

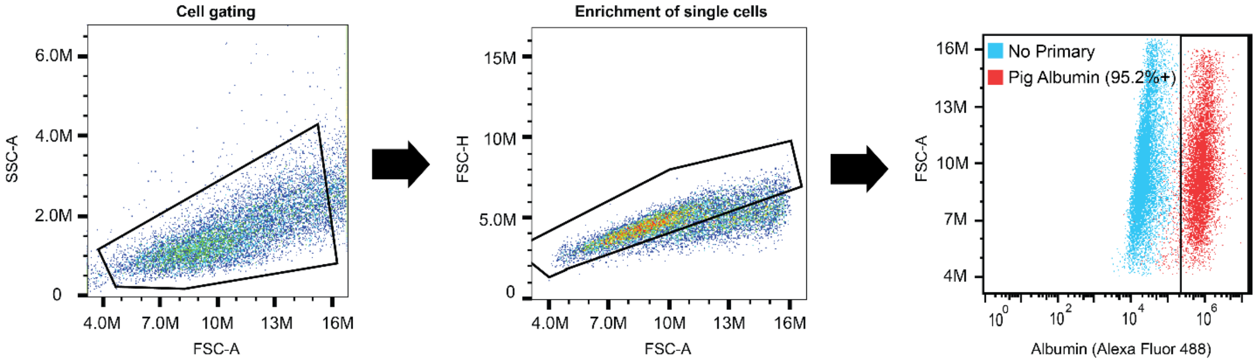

Supplementary Figure S1. Flow cytometry analysis of hepatocyte purity following isolation from a porcine liver. Cells were gated initially based on FSC-A and SSC-A distribution. The population of single cells was subsequently enriched through the exclusion of higher order aggregates on an FSC-A vs. FSC-H plot. Albumin expression of the resulting population was determined by Alexa Fluor 488 fluorescence compared to control cells that were not incubated with the anti-pig albumin primary antibody.

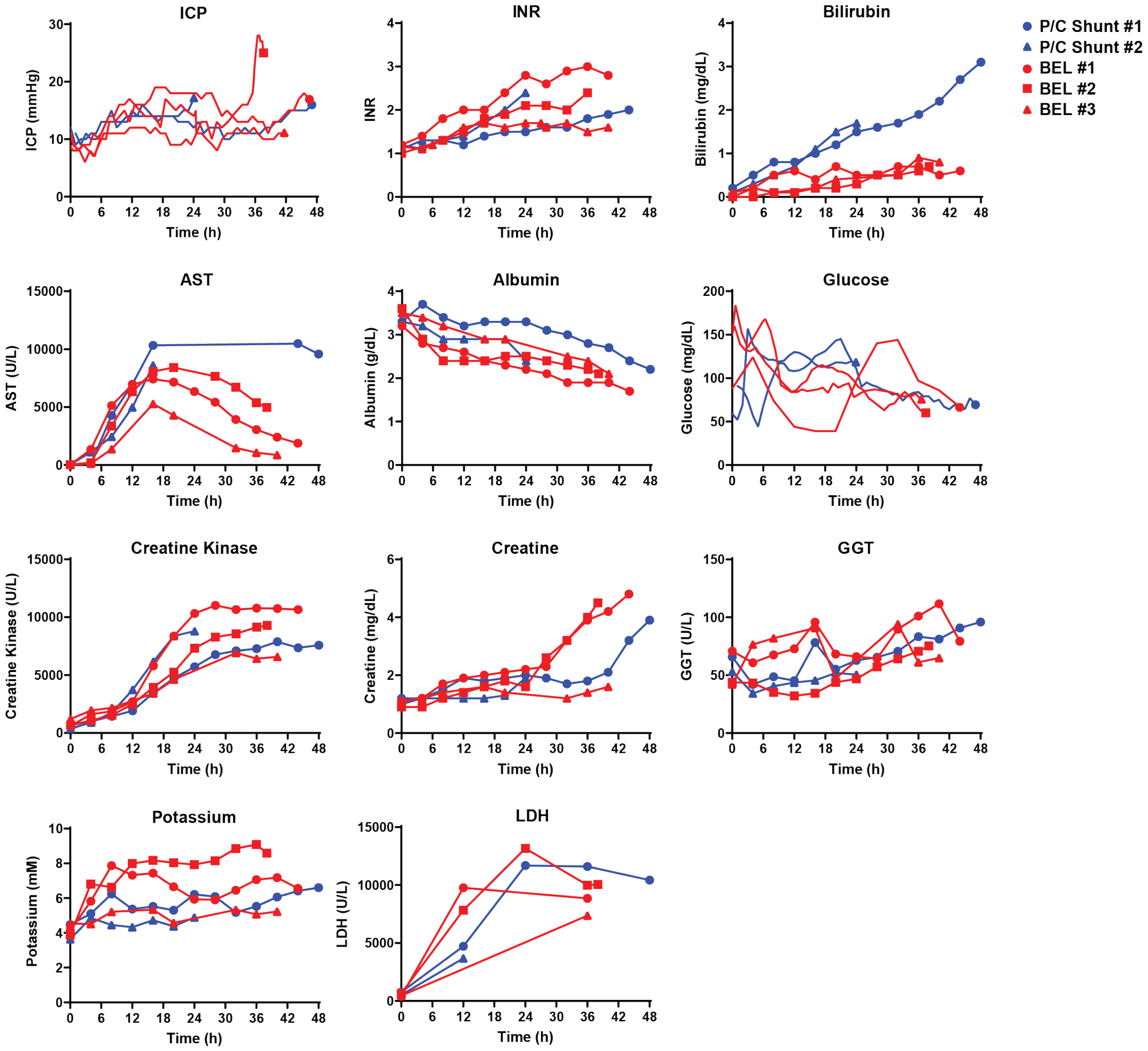

Supplementary Figure S2. Summary of clinical measurements obtained from BEL implant recipient and porto-caval shunt control animals. ICP – intracranial pressure; INR – international normalized ratio; AST – aspartate aminotransferase; GGT – gamma-glutamyl transferase; LDH – lactate dehydrogenase.

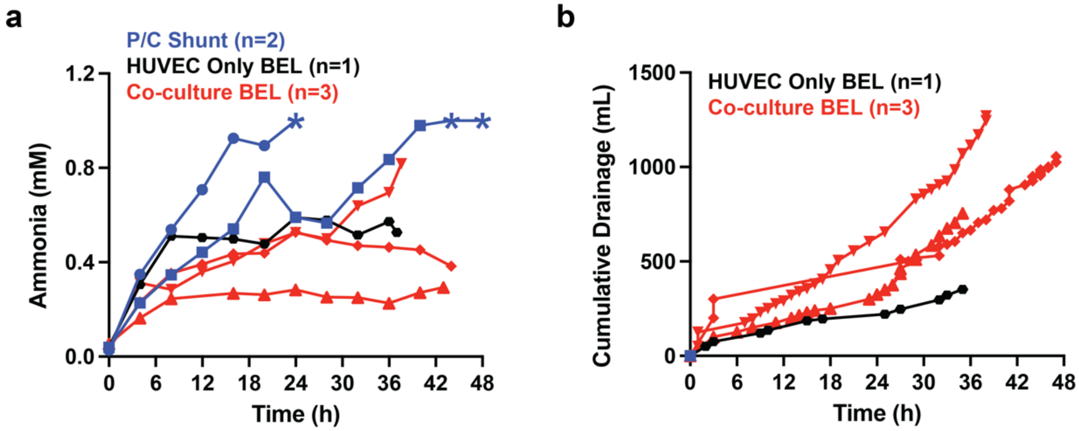

Supplementary Figure S3. Quantification of post-operative blood ammonia levels and cumulative abdominal drainage over time. (**a**) Blood ammonia levels measured post-operatively co-culture BEL and HUVEC only BEL recipient animals, and P/C shunt control animals. (**b**) Cumulative volume of abdominal drainage collected post-operatively from co-culture BEL and HUVEC only BEL recipient animals. P/C – porto-caval; BEL – bioengineered liver.

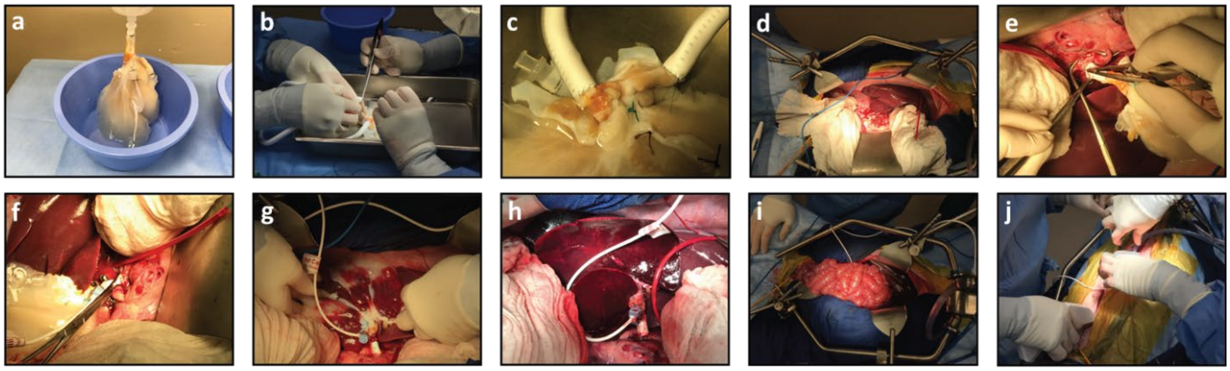

Supplementary Figure S4. Surgical implantation of a BEL. (**a**) BELs are presented to the surgical field and (b-c) 8 mm diameter PTFE prosthetic grafts are anastomosed to the portal vein and iIVC. (**d**) Access is gained through a midline laparotomy and (**e-f**) PV to PV and iIVC to iIVC anastomoses are completed followed by ligation of the native liver. (**g-f**) Perfusion to the BEL is established through the PV and inflow is measured with an ultrasonic perivascular flow probe. (i-j) Once the animals were vitally stable and hemostasis was achieved, an abdominal drain was placed in the surgical field and the abdomen was closed. BEL – bioengineered liver; PTFE – polytetrafluoroethylene; iIVC – infrahepatic inferior vena cava; PV – portal vein

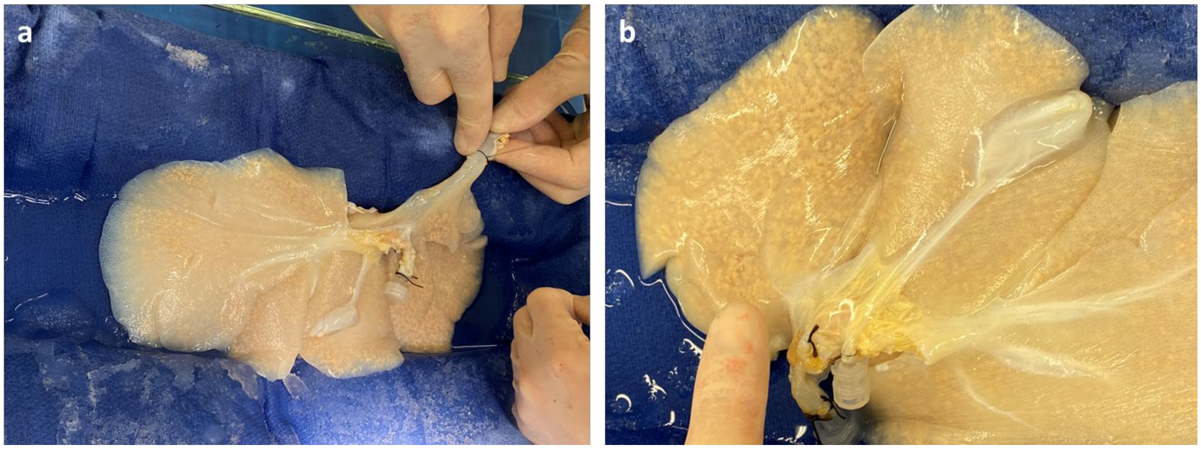

Supplementary Figure S5. Macroscopic cell distribution in a co-culture BEL. (a-b) Representative images showing the distribution of cells visible from the surface of the BEL construct following seeding and culture with HUVECs and porcine hepatocytes.

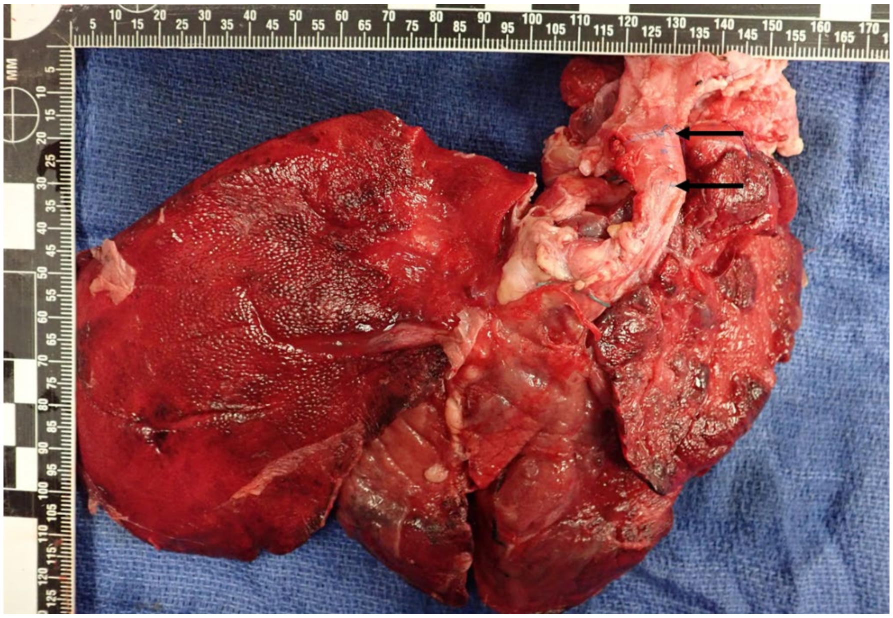

Supplementary Figure S6. Representative image of an explanted BEL following termination. Arrows denote the portal vein anastomosis between the BEL and the native portal vein of the recipient animal.

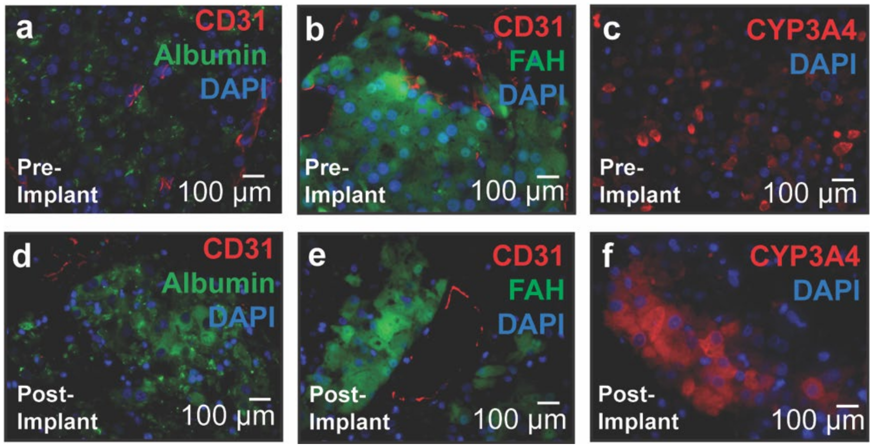

Supplementary Figure S7. High-magnification immunofluorescence of BEL tissue explanted 48 h post-implant. (**a**,**d**) Representative immunostaining BEL tissue (**a**) pre-implant and (**d**) explanted 48 h post-implant showing maintenance of CD31 and albumin expression. (**b**,**e**) Representative immunostaining BEL tissue (**b**) pre-implant and **(e)** explanted 48 h post-implant showing maintenance of CD31 and FAH expression. (**c,f**) Representative immunostaining of BEL tissue (**c**) pre-implant and (**f**) explanted 48 h post-implant demonstrating increased CYP3A4 expression following *in vivo* perfusion.

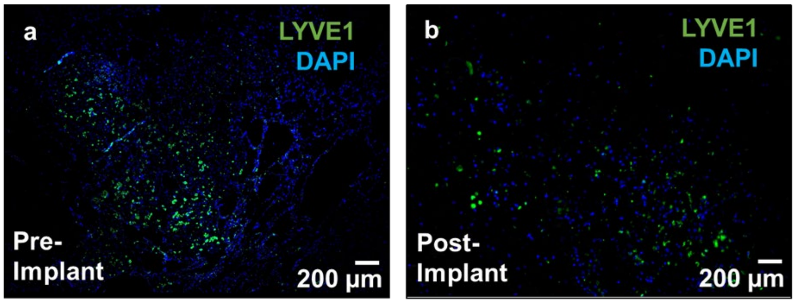

Supplementary Figure S8. LYVE-1 immunofluorescence in BEL tissue explanted 48 h post-implant. (**a**) LYVE-1 expression pre-implant and (**b**) following explant 48 h post-implant demonstrating maintenance of LYVE1 expression.

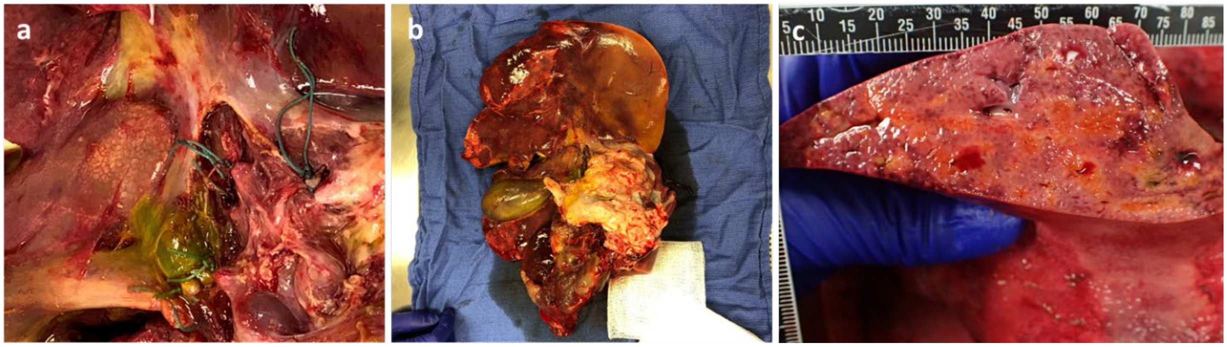

Supplementary Figure S9. Macroscopic images of a devitalized native liver following explant. (**a-b**) Native livers were explanted and (**c**) serially sectioned to assess native liver damage.
