# Supplementary material for: Functional characterization of a bioengineered liver after heterotopic implantation in pigs
Source: Commun Biol. 2021 Oct 7;4:1157. doi: 10.1038/s42003-021-02665-2 (PMC8497596; doi:10.1038/s42003-021-02665-2)
Supplement: Supplementary file 3 — Description of Supplementary Files [file 42003_2021_2665_MOESM3_ESM.pdf]

Description of Additional Supplementary Files

File name: Supplementary Movie 1

Description: Real-time angiogram of BEL perfused with porcine blood.

File name: Supplementary Movie 2

Description: Annotated fly of post-operative anatomy visualized by 3D-reconstruction from CT imaging.

File name: Supplementary Data 1

Description: Microsoft Excel (.xlsx) file containing source data used to generate plots in Figures 2g, 2h, 2j, 2k, 3b, 3c, 4d and 4e.
