# Supplementary material for: Functional characterization of a bioengineered liver after heterotopic implantation in pigs
Source: Commun Biol. 2021 Oct 7;4:1157. doi: 10.1038/s42003-021-02665-2 (PMC8497596; doi:10.1038/s42003-021-02665-2)
Supplement: Supplementary file 7 — Reporting Summary [file 42003_2021_2665_MOESM7_ESM.pdf]

nature research

Corresponding author(s): Scott Nyberg

Last updated by author(s): Aug 7, 2021

Reporting Summary

Nature Research wishes to improve the reproducibility of the work that we publish. This form provides structure for consistency and transparency in reporting. For further information on Nature Research policies, see our Editorial Policies and the Editorial Policy Checklist.

Statistics

For all statistical analyses, confirm that the following items are present in the figure legend, table legend, main text, or Methods section.

n/a	Confirmed	
[ ]	[x]	The exact sample size (*n*) for each experimental group/condition, given as a discrete number and unit of measurement
[ ]	[x]	A statement on whether measurements were taken from distinct samples or whether the same sample was measured repeatedly
[x]	[ ]	The statistical test(s) used AND whether they are one- or two-sided<br>*Only common tests should be described solely by name; describe more complex techniques in the Methods section.*
[x]	[ ]	A description of all covariates tested
[x]	[ ]	A description of any assumptions or corrections, such as tests of normality and adjustment for multiple comparisons
[ ]	[x]	A full description of the statistical parameters including central tendency (e.g. means) or other basic estimates (e.g. regression coefficient) AND variation (e.g. standard deviation) or associated estimates of uncertainty (e.g. confidence intervals)
[x]	[ ]	For null hypothesis testing, the test statistic (e.g. *F*, *t*, *r*) with confidence intervals, effect sizes, degrees of freedom and *P* value noted<br>*Give P values as exact values whenever suitable.*
[x]	[ ]	For Bayesian analysis, information on the choice of priors and Markov chain Monte Carlo settings
[x]	[ ]	For hierarchical and complex designs, identification of the appropriate level for tests and full reporting of outcomes
[x]	[ ]	Estimates of effect sizes (e.g. Cohen's *d*, Pearson's *r*), indicating how they were calculated

Our web collection on statistics for biologists contains articles on many of the points above.

Software and code

Policy information about availability of computer code

Data collection	N/A
Data analysis	GraphPad Prism 8 and FlowJo 10 were used for data analysis.

For manuscripts utilizing custom algorithms or software that are central to the research but not yet described in published literature, software must be made available to editors and reviewers. We strongly encourage code deposition in a community repository (e.g. GitHub). See the Nature Research guidelines for submitting code & software for further information.

Data

Policy information about availability of data

All manuscripts must include a data availability statement. This statement should provide the following information, where applicable:

- Accession codes, unique identifiers, or web links for publicly available datasets
- A list of figures that have associated raw data
- A description of any restrictions on data availability

Source data used to generate the plots in the main figures are included in a supplementary file. Additional datasets analyzed during the study are available from the corresponding author on reasonable request.

Field-specific reporting

Life sciences study design

All studies must disclose on these points even when the disclosure is negative.

Sample size	There was no pre-existing animal model for this type of study therefore this animal model required a significant amount of model development. The sample size was chosen to enable model development and a goal of three surviving animals to demonstrate reproducibility.
Data exclusions	Animals that experienced excessive post-operative bleeding or did not survive the surgical procedure were excluded from the analysis.
Replication	Three successful BEL implant surgeries and two successful porto-caval shunt surgeries were completed and included in the analysis.
Randomization	Allocation was not randomized. Covariates were controlled for by ensuring animals came from the same farm, surgeries were performed at the same site and by the same surgeon as well as ensuring the animals were within the specified weight range.
Blinding	Blinding was not possible for this study since the porto-caval shunt control procedure did not include BEL implantation.

Reporting for specific materials, systems and methods

We require information from authors about some types of materials, experimental systems and methods used in many studies. Here, indicate whether each material, system or method listed is relevant to your study. If you are not sure if a list item applies to your research, read the appropriate section before selecting a response.

Materials & experimental systems

n/a	Involved in the study	
☐	☒	Antibodies
☐	☒	Eukaryotic cell lines
☒	☐	Palaeontology and archaeology
☐	☒	Animals and other organisms
☒	☐	Human research participants
☒	☐	Clinical data
☒	☐	Dual use research of concern

Methods

n/a	Involved in the study	
☒	☐	ChIP-seq
☐	☒	Flow cytometry
☒	☐	MRI-based neuroimaging

Antibodies

Antibodies used

The following antibodies were used for immunofluorescence microscopy:

Primary antibodies:
Rabbit anti-Collagen I (Abcam, AB34710), Rabbit anti-Collagen IV (Abcam, AB6586), Mouse anti-CD31 (Abcam, AB187377), Rabbit anti-Albumin (Abcam, AB79960), Rabbit anti-FAH (Abcam, AB83770), Rabbit anti-Cytochrome P450 3A4 (Abcam, AB3572) and Rabbit anti-LYVE1 (Abcam, AB33682).

Secondary antibodies:
Goat anti-Mouse Alexa Fluor 488 (ThermoFisher, A11029) and Goat anti-Rabbit Alexa Fluor 555 (ThermoFisher, A21429)

The following antibodies were used for flow cytometry:

Primary: Goat anti-pig Albumin (Bethyl A100-110A)
Secondary: Donkey anti-goat Alexa Fluor 488 (Abcam 150129)

Validation

Validation was performed on native tissues to confirm specificity and define appropriate antibody concentrations.

Eukaryotic cell lines

Policy information about cell lines

Cell line source(s)	Human Umbilical Vein Endothelial Cells (HUVECs) were obtained from Lonza.
Authentication	HUVECs (Lonza) were provided with a Certificate of Analysis.
Mycoplasma contamination	All cells were routinely tested for mycoplasma and were confirmed negative.
Commonly misidentified lines (See ICLAC register)	N/A

Animals and other organisms

Policy information about studies involving animals; ARRIVE guidelines recommended for reporting animal research

Laboratory animals	All utilizing live pigs were performed in accordance with the Animal Welfare Act and approved by the institutional animal care and use committee at American Preclinical Services, LLC The pigs used in this study were normal domestic breed swine with a target weight of 30-40 kg.
Wild animals	N/A
Field-collected samples	N/A
Ethics oversight	All studies and protocols were approved and monitored by the Mayo Clinic and American Preclinical Services IACUC.

Note that full information on the approval of the study protocol must also be provided in the manuscript.

Flow Cytometry

Plots

Confirm that:

- [x] The axis labels state the marker and fluorochrome used (e.g. CD4-FITC).
- [x] The axis scales are clearly visible. Include numbers along axes only for bottom left plot of group (a 'group' is an analysis of identical markers).
- [x] All plots are contour plots with outliers or pseudocolor plots.
- [x] A numerical value for number of cells or percentage (with statistics) is provided.

Methodology

Sample preparation	Hepatocytes were isolated from porcine livers through enzymatic digestion with Liberase TM (Roche) and enriched through low speed centrifugation (70 x g). Cell suspensions were fixed in 4% PFA, permeabilized in a buffer containing Triton X100, and labeled with primary antibody goat anti-pig albumin (Bethyl A100-110A) and secondary antibody donkey anti-goat Alexa Fluor 488 (Abcam 150129).
Instrument	BD Accuri C6 Plus
Software	FlowJo 10
Cell population abundance	No cell sorting was performed in the current study.
Gating strategy	Cellular debris were excluded in initial FSC/SSC gating. The population of single cells was further refined by selective gating on a FSC-A/FSC-H projection. The resulting single cell population was measured for Alexa fluor 488 fluorescence to assess the albumin positivity of the population compared to a no-primary antibody control.

- [x] Tick this box to confirm that a figure exemplifying the gating strategy is provided in the Supplementary Information.
